# High-Temperature Oxidation Behaviour of CrSi Coatings on 316 Austenitic Stainless Steel

**DOI:** 10.3390/ma16093533

**Published:** 2023-05-05

**Authors:** Mikdat Gurtaran, Zhenxue Zhang, Xiaoying Li, Hanshan Dong

**Affiliations:** School of Metallurgy and Materials, The University of Birmingham, Birmingham B15 2TT, UK

**Keywords:** CrSi coatings, magnetron sputtering, oxidation, 316 stainless steel

## Abstract

In this study, a closed-field unbalanced magnetron sputtering system, which is environmentally friendly and has high deposition efficiency, was used to deposit CrSi coatings on 316 austenitic stainless steel. This system utilised separate Cr and Si targets, and the appropriate content of Cr and Si of the coatings was adjusted by changing the currents applied to the targets. A series of CrSi coatings with different Si/Cr ratios were produced, and their oxidation behaviour at elevated temperatures was investigated. By analysing the weight gain, surface morphology and microstructure, composition and phase constituents, the oxidation behaviour at 600 °C, 700 °C and 800 °C was investigated and the optimized coating to protect the stainless steel has been identified. The outcome of the research indicated that a small amount of Si (between 4–7 at.%) in Cr coatings is effective in protecting the austenitic stainless steel against oxidation at high temperatures, while a high Si content (around 10 at.% or more) makes the coating more brittle and prone to cracking or delamination during oxidation at 800 °C.

## 1. Introduction

Austenitic stainless steels are materials of choice for many engineering applications, ranging from nuclear power stations to the chemical industry [1,2,3,4], mainly due to their excellent mechanical properties and high corrosion resistance [5,6]. However, the oxidation resistance of austenitic stainless steels needs to be better for such high-temperature applications as rocket engines [7] or the petrochemical industry [8]. In addition, in many high-temperature applications, large temperature change leads to oxide layer spallation because of the thermal expansion differences between the oxide layer formed on the surface and the austenitic stainless steel substrate. Consequently, rapid oxidation of the austenitic stainless steel occurs when it is exposed to an aggressive environment without a protective oxide layer. To extend their service life, the areas of the material in contact with the aggressive environment are made resistant by applying different protective coatings [9,10].

There are many scientific studies on ceramic or metal-based protective coatings applied to stainless steel using different techniques [11,12,13,14]. Metals such as Al, Cr, Ti, and Si are frequently used in protective coatings as their oxides have considerably high oxidation resistance at high temperatures [15,16,17]. Different coatings are applied to increase oxidation resistance at medium and high temperatures [18,19,20]. However, new coatings with desirable oxidation behaviour at high temperatures need to be developed to address the limitations of currently available coatings.

Recently, Cr has drawn increasing attention due to its good antioxidant effect at high temperatures [21,22,23,24]. Characteristically, chromium exhibits good oxidation resistance at high temperatures, with very few crystalline defects that cause an increase in oxidation kinetics [25,26]. However, its use in high-temperature applications is limited due to insufficient mechanical properties causing physical cracking and breakage [27]. In addition, pure Cr coating is completely oxidised at high temperatures [25,28] and does not provide effective oxidation protection unless it has a thickness of more than 11 µm [29,30]. Hence, it has been suggested that a small amount of Si in Cr coatings may improve the oxidation resistance due to the reduction in the oxidation kinetics owing to the single-phase solid solution formed by Si atoms in the CrSi structure [31,32,33]. CrSi coatings have been studied to protect zircaloy 4 [31,32,33,34], Ti-46Al-8N [35], and zirconium-based materials [36] against oxidation at high temperatures.

In the last decade, the production of Cr-based coatings using Cr plating baths has been dramatically reduced and almost fully restricted in Europe due to the formation of toxic chromic acid (known as hexavalent chromium) [37], which is quite hazardous for human health [38]. In this study, a closed-field unbalanced magnetron sputtering system, which is environmentally friendly, was used to deposit CrSi coatings on 316 austenitic stainless steel with a high deposition efficiency. Generally, it is reported in the literature that a CrSi target with a constant Cr and Si composition is used to generate the CrSi coating with fixed composition [27,35,36]. In this study, the closed-field unbalanced magnetron sputtering system utilises separate Cr and Si targets so that the appropriate content of Cr and Si in the coating can be easily adjusted by applying different currents on the targets. In this research, a series of CrSi coatings with different amounts of silicon were produced and their oxidation behaviour at 600–800 °C was investigated. By analysing the weight gain, observing coating morphology and microstructure and studying the composition and phase constituents, the oxidation behaviour of the CrSi coatings was fully studied and the optimised coating used to protect the stainless steel was identified. Application of the CrSi coatings on thermoelectritic materials for oxidation resistance will be carried out and the current study will provide solid background knowledge for further investigation.

## 2. Experimental

### 2.1. Sample Preparation and CrSi Coating Processes

A commercial austenitic stainless-steel rod (Goodfellow, Huntingdon, UK) with a diameter of 25.4 mm was cut (using Struers Accutom-50) to specimens with a thickness of 5 mm. The elemental constituents of the as-received austenitic stainless steel are presented in Table 1. The samples were ground using SiC abrasive papers up to 1200 grit. Before the coating process, the samples were ultrasonically cleaned in acetone and deionised water for 10 min.

CrSi coatings with different compositions were applied to the austenitic stainless steel substrate using the closed-field unbalanced magnetron sputtering technique (Teer Coatings Ltd., Droitwich, UK). Cr and Si targets were placed opposite each other into the physical vapour deposition (PVD) equipment. A constant current of 2 A was applied to the Cr cathode target, while the currents of 0.2, 0.3, and 0.4 A were applied to the Si cathode target to adjust the Si composition in the coatings deposited. The rotation speed of the sample was 5 rpm. The coating process was carried out in three steps: (1) 15 min of ion cleaning of the sample surface using a low current of 0.05 A and 0.2 A for Si and Cr targets under a bias potential of 200 V to remove any contamination; (2) the current of target Cr and Si was ramped up to 0.3/0.4/0.5 A and 2 A in 4-min, and the bias potential was reduced gradually from 200 V to 40 V; (3) deposition of CrSi coating for 35 min at a pressure of 13.33 × 10^−2^ Pa within the argon atmosphere. Due to the ion bombardment, the sample temperature increased slightly in the deposition process to about 100 °C.

### 2.2. Oxidation Testing

A few CrSi-coated samples were oxidised in the air at 600 °C, 700 °C, and 800 °C, respectively, for 80 h, which represented typical operating conditions for many high-temperature applications, using a muffle furnace (SNOL-TMS Europe Ltd., Bradwell, UK) and cooled rapidly (about 50 °C/min) to room temperature in the air environment. Repeated oxidation was taken for the CS4-O8 samples and similar outcomes were confirmed. The weights of the samples were measured using the Ohaus sensitive scale with a sensitivity of 10^−4^ g before and after the oxidation testing.

### 2.3. Microstructure Characterization and Property Evaluation

Systematic microstructure characterisation was performed on both CrSi-coated and oxidised CrSi-coated samples. Surface morphologies and cross-sectional microstructure were investigated using scanning electron microscopy (SEM, Jeol 7000, and Apreo 2 (Jeol-UK Ltd., Welwyn Garden City, UK and Thermofisher Scientific, Stafford, UK), respectively) with EDX devices for chemical concentration and distribution analysis. The surface roughness was also measured using an Ambious XP-200 profilometer (Cntech, Wisbech, UK) with a scanning speed of 0.05 mm/s, over 8 mm length. Proto AXRD diffractometer with a Cu-Kα source (λ = 1.540598 Å) was used to identify the phases of the CrSi coatings. The codes of the samples and corresponding processing parameters are detailed in Table 2. Sample codes of SS, SS-O6, SS-O7, and SS-O8 were given to related stainless steel and oxidised stainless steel samples at 600 °C, 700 °C, and 800 °C, respectively.

## 3. Results

### 3.1. Microstructure of CrSi Coatings

**Figure 1:**
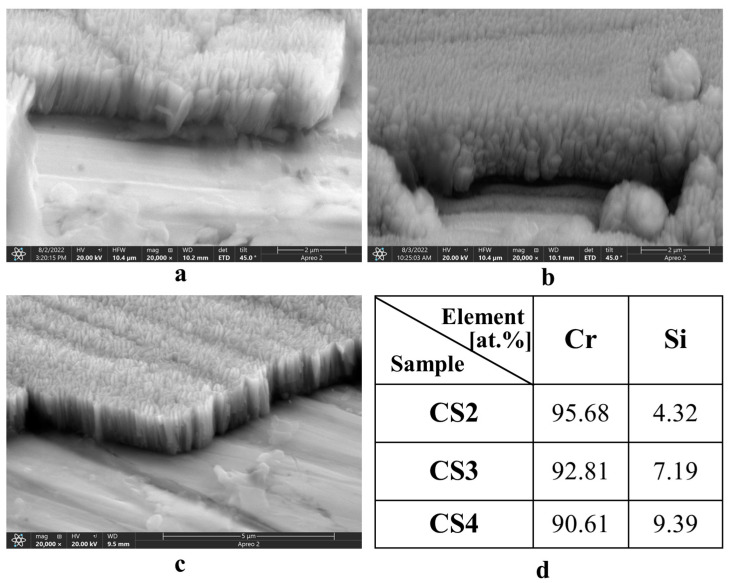
Cross-sectional SEM images of CrSi coating samples (**a**) CS2, (**b**) CS3, (**c**) CS4, and (**d**) EDX analysis of elemental composition (atomic%) for CrSi coating samples.

As shown in Figure 1, the coatings deposited are dense and uniform on the 316 substrates with a fine columnar structure, and the diameter of the column is inversely related to the current applied to the Si target during the coating processes. No pores and spallation were observed on the surface of the coatings. The thickness of the coating layers was around 1.5 µm. EDX analysis of the three CrSi coatings revealed that the atomic composition of Si content increased from 4.32% to 7.16% and 9.39%, corresponding to the Si target current of 0.2 A, 0.3 A, and 0.4 A, respectively, accompanied by the decrease in the column diameter. It implies that the high Si content facilitated the nucleation of the columns. The surface roughness was almost identical for all the coatings, Ra = 0.15 ± 0.010 (μm), confirming the uniform coating structures observed by SEM.

**Figure 2:**
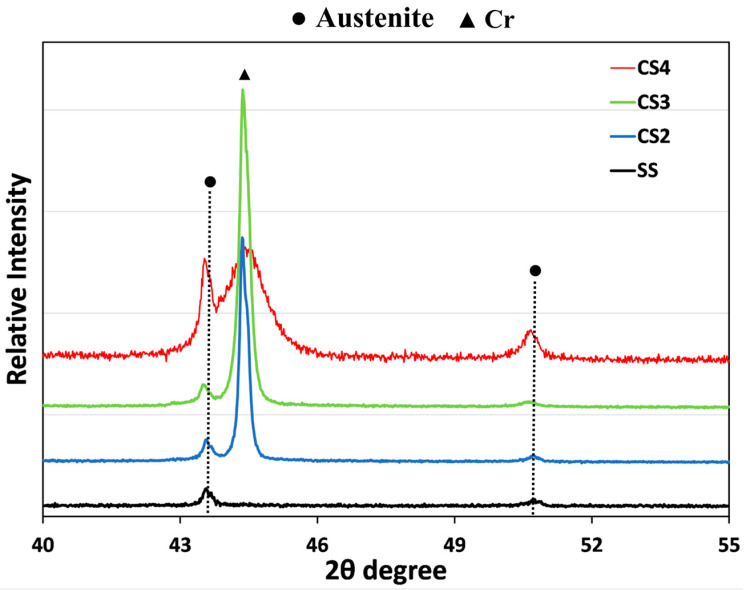
XRD patterns of CrSi coating samples (PDF: 01-085-1335) and AISI316 matrix sample (SS) (PDF: 00-003-1209).

XRD patterns taken from CrSi coating samples are presented in Figure 2, compared with the pattern of the substrate 316 sample. It can be seen that the substrate phase of austenitic stainless steel was detected from all three coating samples, as indicated at 2θ of 43.8° and 51° for (111) and (200) planes. No crystallographic reflection corresponding to the CrSi phases was detected from the coating samples, but still, a dominant (110) signal of reflection corresponding to the Cr-body-centred cubic (bcc) phase was identified, indicating the preferred orientation of the columnar structure with a (110) perpendicular to the surface. It was observed that the position of the (110) signal of reflection with higher Si content was shifted towards a higher angle, and this is due to the decrease in the lattice constant caused by the Si atom in the form of a solid solution. When the Si content reached 9.39 at% (CS4 sample), the intensity of the (110) reduced with a larger full width at half maximum (FWHM), which indicates a distorted crystal structure of Cr and also reduced the preferred columnar growing direction of [110].

### 3.2. Oxidation Behaviour

#### 3.2.1. Weight Gain and Surface Morphology

**Figure 3:**
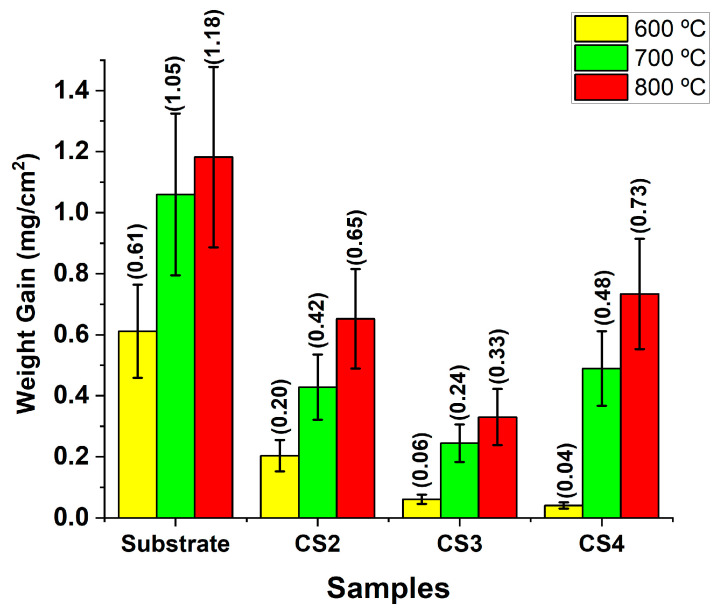
Weight gain of the oxidised CrSi coating samples with the oxidised stainless steel substrate (SS) as a comparison.

Weight gain of stainless steel and CrSi coated samples after oxidation testing at 600 °C, 700 °C, and 800 °C was given in Figure 3. It can be seen that generally, the weight gain changes proportionally to the oxidation temperatures. However, all three CrSi coatings showed a significant reduction in weight gain as compared with the 316 substrate. It can also be seen that the least oxidation weight gain, 0.04 mg/cm^2^, was observed for the CS4-O6 coating sample. When the oxidation temperature was above 700 °C, CS3 coating samples performed the best among all the coating samples with a lower weight gain of 0.24 and 0.33 mg/cm^2^ for 700 °C and 800 °C, respectively.

**Figure 4:**
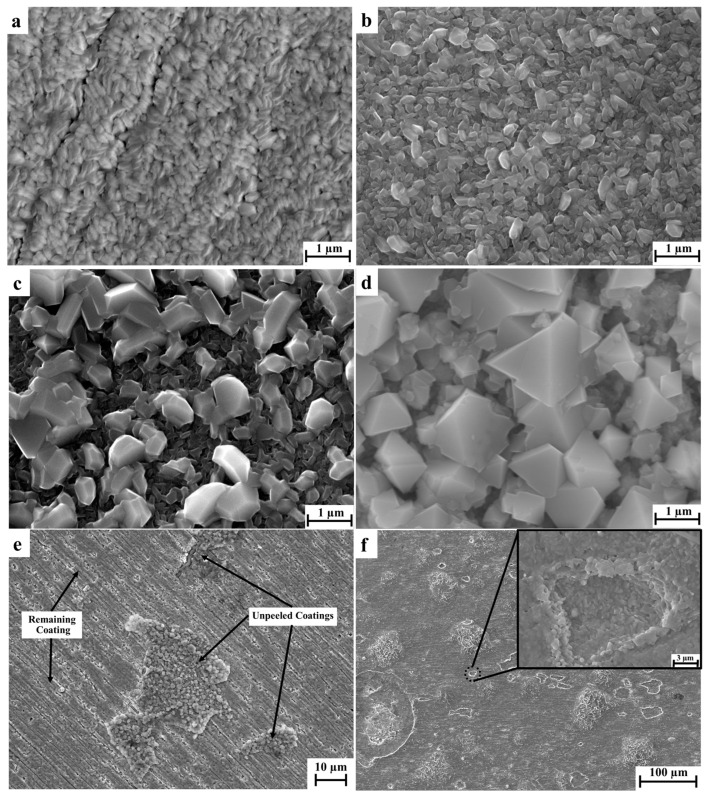
Typical surface morphologies of (**a**) CS3, (**b**) CS3-O6, (**c**) CS3-O7, (**d**) CS4-O8 (high magnification), (**e**) CS4-O8 (low magnification), (**f**) SS-O8.

SEM observation on the surface of oxidised samples revealed similar morphology for the samples oxidised at the same temperatures regardless of the Si contents, and the typical SEM images are shown in Figure 4. It can be seen that after oxidation at a temperature above 600 °C, irregular polygonal oxide particles formed on the surface of the coatings and the higher the temperature, the larger the particles (Figure 4b,c). Some partial spallation was observed for 800 °C oxidised coating samples. However, most of the surface area was still protected by the CrSi coatings (Figure 4e). In contrast, the surface of 316 steel was severely damaged after exposure to 800 °C, as shown in Figure 4f.

#### 3.2.2. Oxide Layer Structure

**Figure 5:**
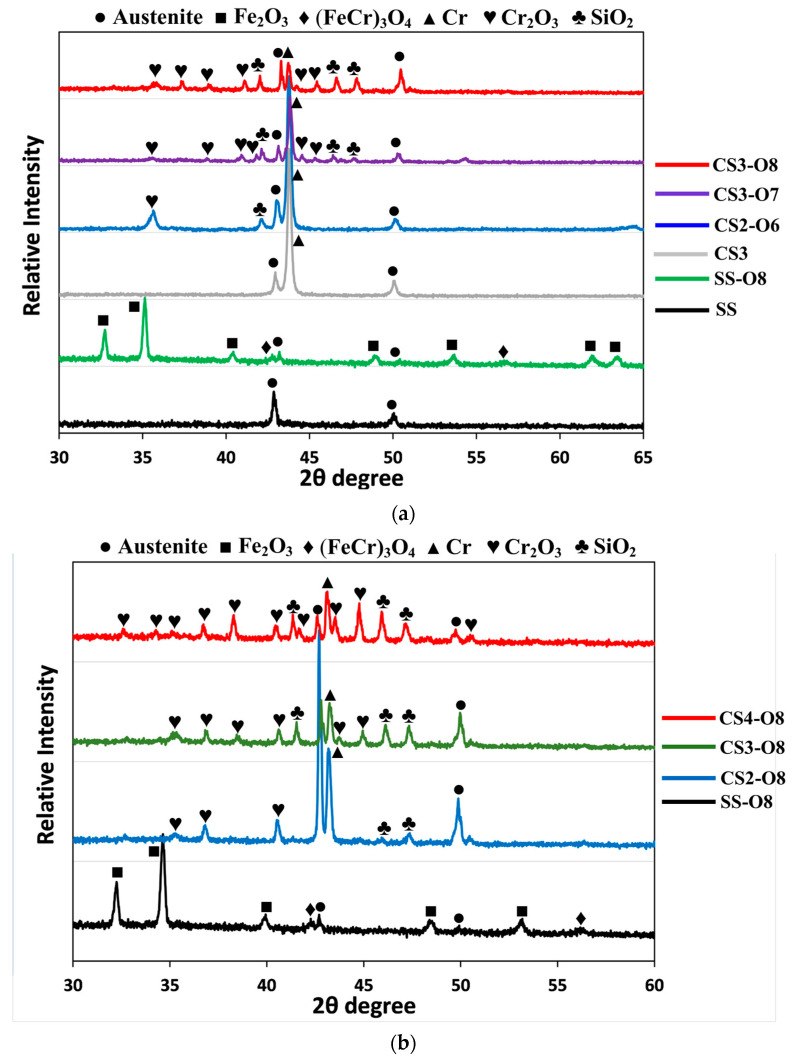
Typical XRD patterns of (**a**) oxidised CrSi coating samples compared with CrSi coating sample, substrate stainless steel and 800 °C oxidised stainless steel sample; and (**b**) 800 °C oxidation treated CrSi samples with different Si content compared with the stainless steel treated at the same condition (PDFs: 00-003-1175, 01-076-1821, 01-076-0938, 01-084-0313).

XRD patterns of the coating samples indicate identical crystallographic reflections when oxidised at the same temperature regardless of the Si content variations. Figure 5 presented typical XRD patterns of the coating samples oxidised at different temperatures compared with XRD patterns of 316 steel and SS-O8 samples. It can be observed that Cr (110) was detected for all oxidised samples, with the intensity of it inversely related to the oxidation temperatures (Figure 5a). A few chromium oxide phases were identified from the CS2-O6 sample. For 700 °C and 800 °C oxidised CS samples, more crystallographic reflections corresponding to chromium and silicide oxides were detected. No signal of reflections of ferrous oxides was detected from all coating samples, indicating oxidation protection of CrSi coatings on the stainless steel surface. The XRD pattern of the 800 °C oxidised stainless steel sample reveals strong (FeCr)_3_O_4_ and Fe_2_O_3_ crystallographic reflections, and the matrix phase of austenitic stainless steel is hard to identify, which implies a thick oxide layer formation on the surface. XRD patterns of different Si content CrSi coating samples after 800 °C oxidation treatment are shown in Figure 5b, with SS-O8 as a comparison. It can be seen that strong intensity and more reflections of the Cr_2_O_3_ and SiO_2_ phases were detected for samples with a higher Si content.

**Figure 6:**
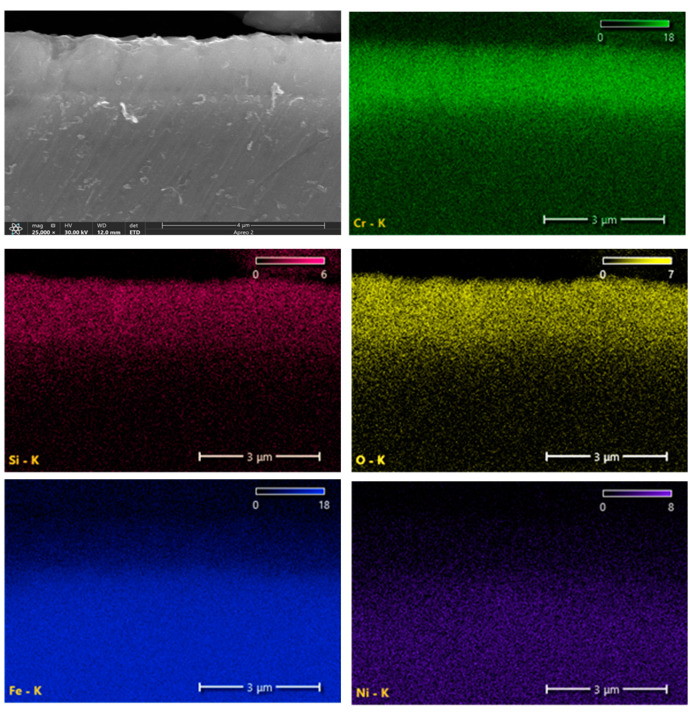
Cross-sectional SEM image of sample CS4-O8 with EDX count-mapping showing elements distributed evenly within the CrSi coating layer.

SEM observation and EDX elements mapping analysis on cross-sectionally prepared oxide layer samples revealed elements distributed evenly within the CrSi coating layer. A typical SEM image, overlaid with EDS maps of Si, Cr, Fe, and O for sample CS4-O8 is shown in Figure 6. It is indicated that neither Si nor Cr were segregated within the coating layer even after 800 °C oxidation.

**Figure 7:**
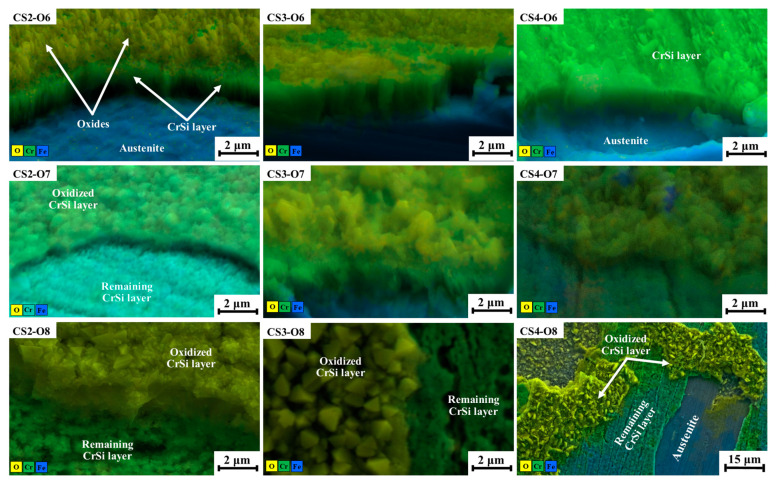
Colour chemi SEM images of fractured oxidised CrSi coating samples as indicated.

Colour Chemi SEM observation on fractured oxidation-treated samples revealed the layer structure differences between the coatings with Si contents. As shown in Figure 7, a thin surface oxide layer formed on CS-O6 samples, and the layer thickness is thicker for lower Si content samples than for the higher ones. Underneath the oxide layer, the CrSi coating layer still adhered to the substrate firmly; no spallation and cracks were found. For 700 °C oxidised CrSi coating samples, the top surface formed a thicker oxide layer, and a few patches of top oxides layer peeling off were observed. However, most formed oxide films protected the austenitic stainless steel surface against oxidation. In particular, the CS3-O7 sample performed less oxidation, as evidenced in Figure 7, where a thick CrSi layer remained on the substrate. However, CrSi coating samples were peeled off of the formed thin surface oxides layer when oxidised at 800 °C; the severity of this phenomenon is related to the Si content of the coatings. For CS2 and CS3 samples, the peeling only happened on the top surface of the formed oxide film, and the CrSi coating layer remained after 80 h oxidation and protected the stainless steel surface from oxidation. However, for sample CS4, which had a coating with a high Si content, not only was the superficial oxide layer that formed on the surface peeled off, but the whole coating layer in some areas was also removed, indicating weak adhesion between the coating and the substrate after 800 °C/80 h oxidation testing when the Si content reached about 10 at.%.

It has also been observed that the oxides formed at 800 °C featured differently as a function of the Si content of the coatings. For low-content Si CS2 samples, the formed oxides are in the shape of round particles of 500 nm in diameter, whereas for medium Si-content CS3 samples, triangular-faceted grains with a diameter of 1200 nm were formed. Further increasing the Si content to about 10 at.%, the formed oxides are mixed with thin rods and round particles in the size of 1700 nm (Figure 7).

## 4. Discussion

A series of CrSi-based coatings with varying Si contents have been observed to withstand damage upon oxidation at high temperatures. As shown in Figure 3, after the oxidation testing, all the CrSi coated samples revealed less weight gain than the stainless steel substrate, which indicates that the CrSi coatings could protect the 316 stainless steel substrate from oxidation. Compared with the oxidation behaviour of the coating samples with different levels of Si concentration, it can be seen from Figure 3 that CS3 and CS4 coatings performed better than CS2 at an oxidation temperature of 600 °C, while CS3 is superior to that CS2 and CS4 coating samples with the lowest weight gain when oxidised at temperatures of 700 °C and 800 °C. The results demonstrate that the extent of the oxidation resistance is Si/Cr ratio related. XRD patterns of the CrSi coatings (Figure 5) confirm that the main phase of the three CrSi coatings (4.3–9.4 at.% Si) is the Cr phase with a body-centred cubic structure. With increasing Si content, the (110) reflection became broader and shifted more to a higher 2 theta angle, or reduced d-spacing of (110), due to the existence of Si atoms in Cr lattices where Si with a smaller radius to replace Cr when in the solid solution. This is in line with the fact that the solubility of Si in Cr is at an atomic concentration between 4.3 and 9.4 (at.%) [39].The decreased lattice constant will induce crystal structure distortion, making the Cr atoms much more difficult to move and delaying chromium oxide formation.

The hypothesis under consideration is substantiated by the SEM images presented in Figure 7, which depict the elemental distribution of oxidised CrSi-coated samples. The revealed thickness of the top surface oxide layer reduces with increasing Si content, except for CS4, when oxidised at 700 °C and 800 °C. This exception may be related to the coating microstructure of CS4. As revealed in Figure 1, the columnar structure of CS4 is much thinner than CS2 and CS3, implying dense columnar boundaries of CS4, which would be the oxygen diffusion tunnels. It is well known that the oxygen diffusion coefficient along grain boundary is 108 times greater than that of bulk diffusion [40]. This is due to the atomic jump frequency in these planar defects, which is about a million times greater than the jump frequency of regular lattice atoms [41]. This fast diffusion of oxygen at high temperatures along columnar boundaries resulted in the worse oxidation resistance of CS4 coating samples. Consequently, oxygen could diffuse to the interface along some loose boundaries and meet with the substrate to form iron oxide, which weakens the adhesion of the CrSi coating to the substrate. This hypothesis was supported by the fact that CS4 coating peeled off more from the substrate than CS2 and CS3 coating samples when oxidised at 800 °C for 80 h (Figure 7). Furthermore, as depicted in Figure 3, the hypothesis under consideration may serve as a probable explanation for the observed weight gain in the CS4-O8 sample. These outcomes are in line with the literature. Zeng, Song et al. [34] suggested that 8% (at.) of Si content in CrSi coatings provides the optimum performance against oxidation at 1200 °C. Similar outcomes were also found by He, Xiujie et al. [36], who stated the superior oxidation behaviour of CrSi coatings with relatively low Si content at high temperatures. Based on the previous studies in the literature and our findings, a further investigation, including oxidation kinetics will be carried out in a follow-up study to see the protectivity of the scale against oxidation. 

## 5. Conclusions

CrSi coatings with different Si concentrations ranging from 4.32 to 9.39 at.% were deposited on 316 stainless-steel substrates using a closed-field unbalanced magnetron sputtering technique. The thickness of all three CrSi coatings is about 1.5 µm and the coating layers are compact with a columnar structure, but the column diameter decreased with increasing the Si concentration.

The oxidation behaviour of the fabricated CrSi coatings at 600 °C, 700 °C, and 800 °C was investigated. Generally, all the coatings possessed good oxidation resistance to protect the 316 stainless steel substrate, and the higher the Si content, the better the oxidation resistance when tested at 600 °C. A superficial oxide layer on the surface of the CrSi coating can prevent further oxidation, thus protecting the stainless steel surfaces from oxidation. A medium amount of Si (7–8 at.%) in the chromium coating (CS3) demonstrated the best oxidation protection of the stainless steel samples when tested at 700 °C and 800 °C. The relatively poorer oxidation protection performance of the CS4 coating, with a high silicon content of 9.4 at.%, is related to the fine columnar structure, which led to fast diffusion of oxygen to the interface between the coating and the substrate, forming iron oxide and thus causing spallation of the coating layer.

Based on these findings, it can be concluded that a small amount of Si (between 4–7 at.%) in Cr coatings is effective in protecting the austenitic stainless steel against oxidation at high temperatures while a high Si content (around 10 at.% or more) makes the coating more brittle and prone to cracking or delamination during oxidation at 800 °C. Further research will be focused on fine microstructure characterization of oxidized CrSi coating to fully understand the oxidation protection mechanism of the coatings with different Si content. Another prospect will be the application of CrSi coatings on different types of substrates, such as thermoelectric materials.

## Figures and Tables

**Table 1:** The chemical composition (at.%) of AISI 316 stainless steel used in this study.

Elements	Carbon	Silicon	Manganese	Chromium	Nickel	Molybdenum	Iron	Other
AISI 316	0.03	1.00	2.00	16.50–18.50	10.00–13.00	2.00–2.50	Bal.	<1.00

**Table 2:** Codes of the samples and corresponding process parameters.

Coating Parameters (Currents)	Sample Codes for CrSi Coatings	Sample Codes for Oxidised Coating Samples		
		600 °C—80 h	700 °C—80 h	800 °C—80 h
		(O6)	(O7)	(O8)
Cr (2 A)	CS2	CS2-O6	CS2-O7	CS2-O8
Si (0.2 A)				
Cr (2 A)	CS3	CS3-O6	CS3-O7	CS3-O8
Si (0.3 A)				
Cr (2 A)	CS4	CS4-O6	CS4-O7	CS4-O8
Si (0.4 A)				

## Data Availability

Not applicable.
